# Supplementary material for: A Low-Glucose Eating Pattern Improves Biomarkers of Postmenopausal Breast Cancer Risk: An Exploratory Secondary Analysis of a Randomized Feasibility Trial
Source: Nutrients. 2021 Dec 16;13(12):4508. doi: 10.3390/nu13124508 (PMC8707938; doi:10.3390/nu13124508)
Supplement: Supplementary file 1 [file nutrients-13-04508-s001.zip › nutrients-1467344-supplementary.pdf]

Table S1. Difference in outcomes from baseline to 16 weeks in low- and high-glucose eating patterns.

	Low-Glucose Eating Pattern		High-Glucose Eating Pattern	
	**Mean Change**	**SD**	**Mean Change**	**SD**
**Weight (kg)**	-7.4	3.9	-5.8	3.7
**Energy intake (kcal)**	-323	410	-445	440
**Fasting glucose (mg/dl)**	-3.3	10.7	-1.5	9.4
**Fasting insulin (µIU/ml)**	-7.0	6.4	-3.5	5.5
**Insulin resistance (HOMA-IR)**	-0.7	0.7	-0.2	0.8
**MAGE (mg/dl)**	-1.8	9.4	2.2	12.6
**CRP (µg/ml)**	-0.5	1.8	-1.4	2.4
**Adiponectin (µg/ml)**	1.8	2.5	-0.9	2.9
**IGF-1 (nM)**	7.6	25.3	12.8	16.0
